# Computational Tools to Facilitate Early Warning of New Emerging Risk Chemicals

**DOI:** 10.3390/toxics12100736

**Published:** 2024-10-12

**Authors:** Farina Tariq, Lutz Ahrens, Nikiforos A. Alygizakis, Karine Audouze, Emilio Benfenati, Pedro N. Carvalho, Ioana Chelcea, Spyros Karakitsios, Achilleas Karakoltzidis, Vikas Kumar, Liadys Mora Lagares, Dimosthenis Sarigiannis, Gianluca Selvestrel, Olivier Taboureau, Katrin Vorkamp, Patrik L. Andersson

**Affiliations:** 1 Department of Chemistry, Umeå University, 901 87 Umeå, Sweden; ioana.chelcea@ri.se; 2 Department of Aquatic Sciences and Assessment, Swedish University of Agricultural Sciences (SLU), 756 51 Uppsala, Sweden; lutz.ahrens@slu.se; 3 Department of Chemistry, National and Kapodistrian University of Athens, 15772 Athens, Greece; nalygizakis@chem.uoa.gr; 4 University Paris Cité, INSERM U1124, 75006 Paris, France; karine.audouze@u-paris.fr (K.A.); olivier.taboureau@u-paris.fr (O.T.); 5 Department of Environmental Health Sciences, Istituto di Ricerche Farmacologiche Mario Negri IRCCS, 20156 Milano, Italy; emilio.benfenati@marionegri.it (E.B.); gianluca.selvestrel@marionegri.it (G.S.); 6 Department of Environmental Science, Aarhus University, 8000 Roskilde, Denmark; pedro.carvalho@envs.au.dk (P.N.C.); kvo@envs.au.dk (K.V.); 7 Department of Chemical and Pharmaceutical Safety, Research Institutes of Sweden (RISE), 103 33 Stockholm, Sweden; 8 HERACLES Research Center on the Exposome and Health, Center for Interdisciplinary Research and Innovation, Aristotle University of Thessaloniki, 54124 Thessaloniki, Greece; spyrosk@auth.gr (S.K.); karakoltzidis.achilleas@gmail.com (A.K.); sarigiannis@auth.gr (D.S.); 9 Environmental Engineering Laboratory, Department of Chemical Engineering, Aristotle University of Thessaloniki, 54124 Thessaloniki, Greece; 10 Environmental Analysis and Management Using Computer Aided Process Engineering (AGACAPE), Institut d’Investigació Sanitària Pere Virgili (IISPV), Universitat Rovira i Virgili (URV), 43204 Reus, Spain; vikas.kumar@urv.cat; 11 German Federal Institute for Risk Assessment (BfR), Max-Dohrn-Str. 8-10, 10589 Berlin, Germany; 12 Laboratory for Cheminformatics, Theory Department, National Institute of Chemistry, 1000 Ljubljana, Slovenia; liadys.moralagares@ki.si; 13 National Hellenic Research Foundation, 11635 Athens, Greece; 14 University School of Advanced Study IUSS, 27100 Pavia, Italy

**Keywords:** early warning system (EWS), new and emerging risk chemicals (NERCs), computational toxicology, risk assessment, artificial intelligence (AI), QSAR, exposure assessment, effect assessment

## Abstract

Innovative tools suitable for chemical risk assessment are being developed in numerous domains, such as non-target chemical analysis, omics, and computational approaches. These methods will also be critical components in an efficient early warning system (EWS) for the identification of potentially hazardous chemicals. Much knowledge is missing for current use chemicals and thus computational methodologies complemented with fast screening techniques will be critical. This paper reviews current computational tools, emphasizing those that are accessible and suitable for the screening of new and emerging risk chemicals (NERCs). The initial step in a computational EWS is an automatic and systematic search for NERCs in literature and database sources including grey literature, patents, experimental data, and various inventories. This step aims at reaching curated molecular structure data along with existing exposure and hazard data. Next, a parallel assessment of exposure and effects will be performed, which will input information into the weighting of an overall hazard score and, finally, the identification of a potential NERC. Several challenges are identified and discussed, such as the integration and scoring of several types of hazard data, ranging from chemical fate and distribution to subtle impacts in specific species and tissues. To conclude, there are many computational systems, and these can be used as a basis for an integrated computational EWS workflow that identifies NERCs automatically.

## 1. Introduction

The chemicals market is global and has an essential role in many aspects of modern life including housing, agriculture and food production, healthcare, and manufacturing of materials and consumer goods. This has led to significant advancements in human welfare, but it also has environmental and human health implications. While some effects have been thoroughly studied by scientists, others are still unknown. The introduction of chemicals on the market and following risks of exposure and effects emphasize how crucial it is to take early measures to recognize and evaluate risks before they are spread. This has also led to initiatives in the European Union (EU) and elsewhere forming a framework for safe and sustainable by design (SSbD) to guide early innovation processes [1].

Given the complex interactions between chemicals and biological systems, protecting the ecological balance and public health requires more than responding to known threats. An early warning system (EWS) is a mean for the prompt detection of new and emerging risk chemicals (NERCs). It would constitute a systematic tool to identify potentially hazardous chemicals, i.e., chemicals that may pose a risk of causing environmental or human health effects. The system should also enable the identification of chemicals posing a risk of exposure through as yet non-explored emission sources, or because of changed or increased use. It should enable the identification of newly introduced chemicals but also of known chemicals with, e.g., new use patterns or newly discovered hazard properties. In this context, a European EWS framework has recently been proposed by the European Commission as a key component of the Chemicals Strategy for Sustainability (environment.ec.europa.eu).

An EWS is an integrated system for monitoring and collecting data, and analyzing, interpreting, and communicating data, which can be used to make decisions early enough to protect humans and the environment [2]. An early warning is, for example, critical to ensure efficient management in the healthcare sector, for tracking, predicting, and quickly reacting to disease outbreaks and health emergencies [3,4,5]. Additionally, these systems have been used to anticipate and prevent disasters that range from chemical spills [6] to natural disasters [7], allowing for containment measures or rapid evacuation. EWSs have also been utilized in environmental conservation to detect signal changes in ecological parameters, allowing for prompt actions for safeguarding natural resources [8]. The evolution of EWSs across these distinct domains demonstrates their adaptability and critical role in dealing with and preventing hazards and disruptions.

Chemical EWSs can take on various degrees of complexity from a transparent human expert-driven approach where signals of NERCs are identified by individual experts using different data sources, to chemistry- or biology-driven experimental approaches towards computational methods. Chemistry-driven methods to identify NERCs cover advanced analytical techniques and are often based on mass spectrometry, including target, suspect screening (SS) and non-target screening (NTS) approaches. These methods can deliver detection, and in some cases, quantification of chemicals in a variety of matrices, such as food, abiotic and biotic environmental samples, materials and consumer goods, and human tissue samples. Several biology-driven experimental techniques, such as effect-directed analysis (EDA) and effect-based monitoring (EBM), aim at detecting chemical hazards promptly. These techniques are designed to evaluate biological signals induced by individual or complex mixtures of chemicals in environmental samples, such as soil, water, and air [9]. The sensitivity and wide detection range of these bioassays make them suitable for screening known and unknown substances. To identify potentially hazardous chemicals, EDA integrates a biological effects assessment with SS and NTS applying advanced chemical analysis [10]. Sample extracts are fractionated and tested to find the most bioactive fractions, and these are analyzed aiming to identify chemicals causing the observed effects. A few EWSs are in operation including national initiatives, such as the expert-based system under the Swedish Toxicological Council (kemi.se) and the German water-based system focused on NTS data and EBM led by the German Environment Agency (umweltbundesamt.de). In addition, the NORMAN network for identifying hazardous chemicals in the environment [11] has an EWS initiative focusing on use of SS/NTS [12].

Experimental methods in EWSs have several drawbacks including a dependence on skilled staff, representative sampling, and costly equipment. A computer-driven EWS is an alternative, providing faster and scalable solutions at a reasonable cost. These systems can also be adaptable, considering big datasets, conditions that change over time and analyze chemicals lacking analytical standards, and even those not yet on the market. Another key driver of using predictive hazard modeling is the possibility to reduce ethically questionable animal testing. This follows the 3Rs principle to reduce, refine, and replace animal testing [13] which have enabled forecasting important endpoints, such as endocrine disruption, skin sensitization, and mutagenicity.

Computational tools are also critical for developing a next-generation risk assessment (NGRA) and new approach methodologies (NAMs) [14,15]. Several kinds of computational tools exist, including natural language processing (NLP) methodologies, which could serve as a significant component by efficiently identifying and structuring relevant data [16,17]. Additionally, quantitative and qualitative models, including quantitative structure–activity relationship models (QSARs) [18], can be applied to derive both hazard and exposure data [19,20]. Furthermore, incorporating bioinformatics methodologies and systems biology expands the scope of biological data for EWS applications [14,21]. An EWS also benefits from unsupervised methods including clustering and rule-based approaches [22,23]. These techniques enable the identification of commonalities between known and unknown potentially hazardous chemicals. Finally, a reliable EWS should consider the entire “source-to-dose” continuum. This includes robust exposure assessments, environmental multimedia modeling, and the use of physiologically based kinetic (PBK) models to anticipate the internal dose [20,24]. It is important to remember that computational methods rely on experimental data and may have limited applicability domains depending on the methodology and training data.

A computer-driven chemical EWS requires data as a trigger that could stem from, e.g., large databases, repositories of scientific literature, patent databases, or monitoring and screening campaigns. These data sources may offer a multitude of insights into chemical properties, usage trends, and various hazard measures. Chemicals with data that signal a potential hazard or fate property of concern, increased use or abundance in products or environments, necessitate further analysis. Computational and predictive methods offer an opening to assess the risk of exposure and effects, and those data could be combined and synthesized providing signal strength for the notification of a potential NERC.

In this paper, we present the current state of knowledge regarding computational methodologies applicable for an EWS tool, providing an understanding of the opportunities and challenges in the development and implementation of a computational EWS. We also outline the strengths along with the weaknesses of pre-existing computational modules for use in an EWS and describe general-purpose and easily implementable computational tools suitable for an automated EWS workflow. Additionally, we highlight potential challenges in developing an efficient automated computational EWS using the most recent technologies including artificial intelligence (AI).

## 2. Structure Curation and Data Sources

### 2.1. Structure Curation

An EWS requires accurate and readable information linking a chemical structure to physicochemical properties and hazard data. Although Chemical Abstract Service (CAS) numbers serve as distinctive identifiers, they cannot describe the chemical structure, as the same compound may be associated with multiple CAS numbers. Structural information can be extracted from CAS numbers using the US-EPA Chemistry Dashboard for Python (cirpy.readthedocs.io) or the Chemical Identifier Resolver (CIR) (cactus.nci.nih.gov/chemical/structure) in KNIME [25]. The Simplified Molecular Input Line Entry System (SMILES) is frequently used to represent the chemical structure of a compound, but it lacks 3D information, and it may increase the risk of duplication. In contrast, InChIKeys offer precise information on the chemical structure and can only be presented in a specific format, making them useful for identifying duplicates. Overall curation is critical, and structure format must be tailored for applied computational hazard models.

### 2.2. Data Sources

Scanning existing and emerging databases or other open sources is a starting point of a computational EWS relying on robust and well curated data. These could cover databases of experimental data to inventories of chemical properties, usage, and environmental and human health impacts. Recently, over 900 databases were reviewed and classified into 13 different types, including information on physicochemical properties, toxicological information, omics data, product and material usage and characteristics, patents, environmental and human monitoring data, and adsorption, distribution, metabolism, and excretion (ADME) [16]. Large chemical registry databases are instrumental in determining new compounds including, e.g., ECHA (echa.europa.eu), PubChem (pubchem.ncbi.nlm.nih.gov), ChEMBL (ebi.ac.uk/chembl), ChemSpider (chemspider.com), and CAS SciFinder (cas.org). Another invaluable resource is the EPA CompTox Dashboard (comptox.epa.gov/dashboard), which has over 1.2 million entries with information on chemical structures, experimental features, and toxic effects. Databases such as the US EPA’s IRIS (epa.gov/iris) and the ECOTOX database (cfpub.epa.gov/ecotox) offer species-specific toxicity information along with reference values for environmental toxicity. Emerging databases include those compiling omics data, such as ArrayExpress (ebi.ac.uk/biostudies/arrayexpress) and BiGG (bigg.ucsd.edu). The ACToR database (actor.epa.gov) consolidates data on environmentally significant chemicals from over 400 different databases and datasets. The CEBS database (manticore.niehs.nih.gov/cebssearch) compiles animal experimental data from the U.S. National Toxicology Program (NTP), offering both general biological information and toxicological data. The Comparative Toxicogenomics Database (CTD) (ctdbase.org) integrates data on associations between chemicals, gene products, phenotypes, diseases, and environmental exposures. It provides insights into interactions, such as chemical–gene, chemical–phenotype, chemical–disease, gene–disease, and chemical–exposure relationships.

Patent inventories could also be important including Derwent World Patents Index (DWPI) (clarivate.com), SureChEMBL Beta (surechembl.org), European Patent Office (epo.org), USPTO (uspto.gov)), and certain national registry databases [26]. Patents can be an opening for early identification of NERCs even before they are commercialized by anticipating possible sources and exposure pathways. To provide a comprehensive understanding of substance monitoring, the European Union’s Human Biomonitoring (HBM) Dashboard (hbm4eu.eu), the IPChem Portal (ipchem.jrc.ec.europa.eu), and the NORMAN Network (norman-network.com) provide examples of large databases on a variety of compounds across matrices, including food, consumer goods, environmental samples, and human tissues.

Chemical scientific literature and grey literature, including stakeholder reports and social media, emerge as additional vital sources of information. NLP can be used to extract information from such sources, and tools like ExaCT [27], EPPI Reviewer [28], and Robot Reviewer (robotreviewer.net) are designed to automatically extract data from scientific literature. Another example is AOP-helpFinder (aop-helpfinder), which is designed to identify chemical–biological event and event–event relationships in scientific articles, notably within databases like PubMed [29]. In addition to curated chemical databases, high resolution mass spectrometry (HRMS) analysis generates large datasets that capture a wide range of chemicals in samples, according to the analytical procedures and instruments used. Digital advancements such as the Application Programming Interface (API) on a Digital Sample Freezing Platform (dsfp.norman-data.eu) for HRMS data enable automated retrieval of exposure data as well as semi-quantification of chemical concentration levels. 

## 3. Exposure Models

To identify NERCs, it is important to comprehend how emerging chemicals are being spread and distributed in key environmental media, and how they may reach humans and target tissues in humans. This will form data on the exposure potential of chemicals and the most significant pathways. Numerous computational tools are available for different matrices and pathways to determine the external or internal exposure of emerging contaminants.

### 3.1. External Exposure Models

#### 3.1.1. Human External Exposure Models

For an EWS, high-throughput exposure models could be appropriate for the assessment of human exposure as they are generic and capable of covering a variety of exposure routes, and due to their ease of integration into an EWS workflow [30]. For exposure through indoor environments, the SHEDS-HT model provides a chemical screening capability with few parameters required and including different exposure routes [20]. Another actively maintained indoor exposure model, RAIDAR-ICE, has been modified for use in Excel and is suitable for screening. It includes a PBK model for different exposure routes [24]. Likewise, several exposure scenarios are included in the EUSES tool (echa.europa.eu), such as SimpleBox for environmental multi-media fate modeling and ConsExpo for consumer exposure. Although some models have been developed to predict occupational exposure for a specific exposure pathway, their applicability to screen multiple compounds in batch is still limited [19].

Using individual consumption data from the EFSA Comprehensive European Food Consumption Database, EFSA developed the Dietary EXposure (DietEX) tool to calculate dietary exposure to substances present in food (efsa.europa.eu). The tool estimates the mean and the 95th percentile exposure for various age classes and specific population groups in several EU countries. Similarly, the Rapid Assessment of Contaminant Exposure (RACE) tool compares the results to the health-based guidance value or other pertinent toxicological reference values and provides exposure estimates (mean, median, and 95th percentile) of various population groups to chemical contaminants that originate from single food items. The key difference between the two tools is that RACE can only estimate exposure to a single food item at a time, whereas DietEX can estimate exposure to multiple foods. Moreover, DietEX does not share RACE’s scope limitation of only including chemicals that have previously undergone EFSA assessment. However, RACE assesses and categorizes the related risks, whereas DietEX is only intended for exposure estimation.

#### 3.1.2. Environmental Fate Models

Fate models evaluate the environmental distribution of a compound by calculating the distribution among specific compartments including water, soil, air, and sediment. This information can then be utilized to estimate the predicted environmental concentration (PEC). Fate models can take on global, regional, and local scales, and have been developed for describing atmospheric or multi-media transport, including software platforms such as INTEGRA [31], SoilPCA [32], EpiSuite, BETR North America [33], NEM [34], SimpleBox [35], CoZMo-POP [36], USEtox [37], Merlin-Expo tool [17], and the PiFs model [38]. Fate modeling requires data on the characteristics of the environment and chemical properties including persistence. EpiSuite (epa.gov) can be used to derive persistence measures, although this lacks information on the applicability domain and does not differentiate between persistence in different environmental matrices. The VEGA platform (vegahub.eu) provides a range of both quantitative and qualitative models of persistence for soil, water, air, and sediment. Predictions from VEGA include an estimate of reliability based on the model’s applicability domain that could be used in scoring exposure and hazard reliability and impact. The chemical fate is to a large extent determined by intrinsic physicochemical properties including water solubility, vapor pressure, and partitioning coefficient between organic matter and water [39]. EpiSuite and VEGA offer predictive models for these properties.

### 3.2. Internal Exposure Models

Models that predict internal concentrations in organisms are useful tools for obtaining more detailed insights into chemical risks. They can provide estimations of internal concentrations or even doses at the target of toxicity. These measures can be calculated using a variety of methods, covering predictive models for bioconcentration or bioaccumulation, and organism-specific compartmental models.

#### 3.2.1. Bioconcentration and Bioaccumulation Models

In aquatic organisms, bioaccumulation is usually reported in metrics such as bioconcentration factors (BCFs), bioaccumulation factors (BAFs), or biomagnification factors (BMFs). However, for some terrestrial organisms, e.g., earthworms, biota-to-soil accumulation factors are reported [40]. These factors are usually calculated using empirical data or models that consider both the organism characteristics and the compounds’ physicochemical properties. There are few empirical models for predicting BCFs in species other than fish due to a lack of experimental data for model building [41]. Additionally, chemical applicability is also limited, with present models focusing primarily on non-ionic organic compounds. The BCFBAF model in EpiSuite estimates these properties by either applying a linear regression model utilizing the logarithm of the octanol–water partitioning coefficient (*K_ow_*) to empirical data or combining *K_ow_* with predicted metabolic half-lives in fish as in the Arnot–Gobas method [42]. The VEGA platform includes four BCF models: CAESAR, Meylan, Arnot-Gobas, and KNN-Read-across, and provides a reliability score and the six most similar substances within the training data (vegahub.eu). Both the EpiSuite and the VEGA BCF models have been used in EWS and NERC prioritization, allowing a relatively rapid calculation of data for many chemicals [43]. Empirical bioconcentration and bioaccumulation models may be insufficient for predicting internal exposure to emerging compounds because they do not consider the species-specific physiology or ADME properties, and, in addition, they only provide an estimate of the whole-body concentration rather than specific target organs of toxicity.

#### 3.2.2. Compartmental Models

Internal concentrations in organisms can be predicted using basic one-compartment models treating the organism as a single compartment with a consistent chemical distribution throughout the organism. They facilitate an opening for fast screening, one example being the high throughput toxicokinetic (HTTK) package by US-EPA featuring both a one-compartment and a three-compartment model for hundreds of different compounds to simulate internal exposure in humans, rats, mice, rabbits, and dogs (httk). Furthermore, Wiecek et al. present a generic human one-compartment model and PBK model with the goal of carrying out forward dose measurement for a human health risk assessment of chemicals in food [44]. The primary challenge relates to the availability of data for metabolic parameters that require in vitro measurement. Hendriks et al. presented a compartmental model that uses *K_ow_* and a few species-specific parameters to simulate the build-up of chemicals for various trophic levels [45].

#### 3.2.3. Physiologically Based Kinetic Models

PBK models provide a useful computational tool for estimating internal concentrations, the dose at target, and understanding the ADME of chemicals. Additionally, quantitative in vitro-to-in vivo extrapolation using PBK models could be used to reconstruct exposure and generate non-animal-based data for risk assessments [46,47]. A recent overview of PBK models revealed significant knowledge gaps in their chemical applicability domain and concluded that most are created for low molecular weight compounds, which typically follow Lipinski’s rule of five [48]. Several governmental agencies have set the objective for the next-generation PBK models to develop these without the use of in vivo data [49]. A large portion of the parameterization can be accomplished using in vitro and in silico data. A range of compound-specific and generic models, such as INTEGRA [31], MENTOR-3P [50], and the MERLIN-Expo tool [51], are available. Generic PBK models are available, e.g., for fish species [52] and farm animals [53].

Tebby et al. concluded that models relying on pre-existing databases or basic QSAR models for parametrization are practical and applicable for screening and lower-tier calculations [54]. PBK models have also been combined with effect-based safety limits to determine which subgroups and what percentage of the population are subjected to exposure levels above safety limits [49,55,56]. One such model is the lifetime PBK model, which was created to examine the effects of PFAS compounds on humans [57]. Another option is using the HTTK package to simulate population kinetics with pre-defined physiological parameter distributions [55,56]. Overall, PBK modeling could be used for screening; however, the models require extensive parametrization and are mostly compound-specific rather than generic. Therefore, further development is needed for their use in EWS. One of the major challenges with the parameterization of PBK models is the need for compound-specific biotransformation data.

#### 3.2.4. Biotransformation Models

Most in silico biotransformation models are designed for pharmaceuticals, making them less well suited for application on industrial chemicals. In addition, large individual and interspecies variability in metabolic enzymes make it challenging to develop models for predicting biotransformation parameters, such as intrinsic clearance rates. Primary biotransformation half-lives and rate constants in fish can be predicted using the half-life model included in the VEGA platform [58]. However, models for other species are lacking, indicating a significant data gap and the need to develop new tools. The OECD QSAR toolbox [59], CTS (qed.epa.gov/cts), BioTransformer [60], and EAWAG-BBD/PPS [61] are examples of open-source software aimed at predicting transformation products [62]. An example of available commercial software is Meteor Nexus [63]. The software CTS, EAWAG-BBD/PPS, and Meteor Nexus offer likelihoods of formation of a given transformation product, whereas the other models only predict formed products. Degradation in the environment can also be evaluated using two VEGA models that predict ready biodegradability. Additionally, the JANUS tool automatically generates environmental degradation products (using over 200 degradation pathways) and predicts degradation products (vegahub.eu).

## 4. Effect Models

Comprehending a chemical’s ability to cause effects in organisms and its mechanism of action are key components in assessing the hazards of chemicals. However, understanding which effects may pose a hazard and lead to adverse outcomes requires a contextual framework such as the one provided by adverse outcome pathways (AOPs) [64]. AOPs are a means to systematize and organize pathways leading to adverse effects initiated by a molecular initiating event (MIE) triggered by a stressor (e.g., chemical), and continuing through one or several key events (KE). AOPs are today constructed for many health effects (aopwiki.org) and efforts are being made to build quantitative AOPs (qAOPs) and adverse outcome networks, and to include the concept in risk assessment processes [14,15]. Computational effect models in the form of QSARs are therefore oftentimes developed to predict MIEs and KEs. In addition, the rapid development of bioinformatics tools for the analysis of omics data will enable using such data in systems biology approaches to understand chemical-induced perturbations leading to systemic effects.

### 4.1. Quantitative Structure-Activity/Property Relationship Models

QSARs and quantitative structure property relationships (QSPRs) have been used to quickly screen substances and provide both biological activity and chemical property values for a wide range of endpoints and substances [65]. Inventories exist where models have been collected (life-concertreach.eu) and certain tools can predict various properties by integrating multiple models. Several of these tools, such as QsarDB (qsardb.org), VEGA (vegahub.eu), EPISuite (epi-suite), QSAR TOOLBOX (qsartoolbox.org), and OPERA (ntp.niehs.nih.gov), are free to use. In addition, the Danish (Q)SAR Database provides predictions from a large range of models (qsar.food.dtu.dk). JANUS (vegahub.eu) is primarily designed for prioritization and it is accessible through the VEGAHUB platform, providing both predicted property values and experimental data for a range of substances processed in batch. The models implemented in JANUS refer to REACH requirements and thresholds, covering critical endpoints such as carcinogenicity, mutagenicity and reprotoxicity (CMR), persistence, bioaccumulation, and toxicity (PBT), and endocrine disruption. In the VEGA tool there are currently 112 distinct models predicting almost 50 properties covering environmental fate and distribution, toxicokinetics, human toxicity, and ecotoxicity.

The validity of applied models is critical both for deriving sound data but also for regulatory acceptance [66,67]. In the development of QSAR models it is important to wisely select training and test data sets, and to report model parameters, settings, and outcomes in a transparent way, e.g., using the QSAR model reporting format [68]. The OECD validation principles were derived to increase the use and acceptance of QSAR models urging modelers to include information not only on algorithm, endpoint information, and performance statistics of models but also on a defined domain of applicability [67]. To ensure the accuracy and applicability of its predictions, the third OECD principle states that a QSAR model should only make predictions inside the chemical space on which it has been trained and verified. Today several models offer this evaluation automatically. The evaluation of the applicability domain can be qualitative (inside or outside) or quantitative, i.e., with a continuous value. The use of quantitative values offers advantages including (1) allowing comparisons of results from several models, and (2) integrating results from multiple models assigning specific weights based on the applicability domain value.

### 4.2. Complementary Computational Tools

Big data is generated by emerging omics technologies and bioinformatics network sciences, which enables the evaluation of interactions between chemical exposure, gene expressions, pathways, and adverse outcomes. The biological mechanisms underlying toxicity endpoints and/or toxicity biomarkers can be inferred from differentially expressed genes. One of the primary sources of integrated toxicogenomics data, which enables scientists to assess effects of toxicants based on gene expression, is ToxicoDb [69]. The ability to pinpoint precise molecular pathways and mechanisms that a chemical may affect is one benefit of leveraging omics data [70]. However, data produced using omics technologies may be intricate and challenging to understand, necessitating the use of sophisticated analytical bioinformatics tools and field expertise. They can help understand the uncertainty, temporal trends, and possible health risks related to chemical exposure. Another option would be to apply systems biology models that are designed to replicate the intricate relationships that exist between various biological systems and processes [71]. These mathematical models combine metabolic control analysis, flux balance analysis, and elementary model analysis and could be used to comprehend network-associated toxicity pathways.

Molecular dynamics (MD) simulations aim to analyze a chemical’s interaction with target receptors, i.e., MIEs. These calculations, while powerful, are computationally expensive and require a deep understanding and three-dimensional structures of both the receptors and the target compounds [72,73]. However, their utility goes beyond mere analysis by providing detailed insights into the molecular behavior and helping with the mechanistic interpretation of the endpoint under study. In addition to molecular dynamics simulations, molecular docking serves as a complementary approach in EWSs for risk assessment [74]. Molecular docking focuses on predicting the binding affinity and orientation of small molecules within the binding site of a target receptor. This method is less computationally expensive and can be applied to large libraries of compounds, making it an attractive alternative, especially in high-throughput screening scenarios. While molecular docking may not provide the same level of detailed insight into molecular interactions, its ability to handle large datasets quickly and efficiently makes it a valuable tool in risk assessment, especially in situations requiring rapid screening.

## 5. Data Integration

**Figure 1:**
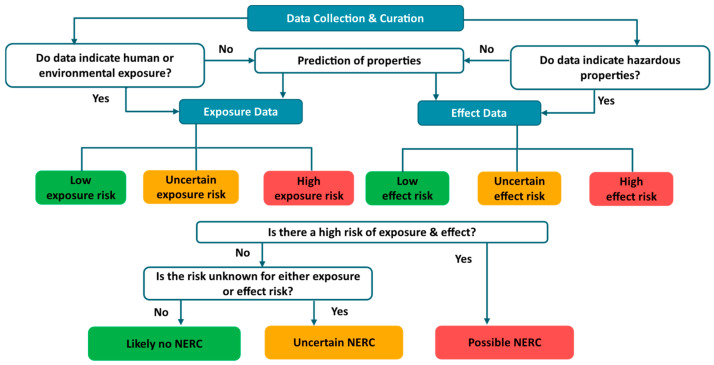
An example decision tree that classifies substances into three main groups—I. Possible NERC, II. Uncertain NERC, and III. Likely no NERC—using EWS data integration.

Data generated in the exposure and effect modules will be integrated into an EWS framework aimed at identifying and flagging potential NERCs. It is critical that this process is based on high-quality data ultimately following the FAIR (findable, accessible, interoperable and re-usable) principles [75] if using data inventories, or based on well-curated chemical structures and sound models if data are estimated. However, the quality of compiled estimated and experimental data will differ, and they might also contain inaccurate data as, for example, big data from multiple databases could be heterogenous. It will thus be crucial to examine data quality and spot any possible anomalies. To analyze the various data types, expert judgment will be required for, e.g., setting weighting factors and selecting parameters. Simultaneously, a high degree of automation should be implemented in the process to allow for a quick and unbiased identification of NERCs from big data. Reliability weights and scores associated with the applicability domain of models, if provided, is one opening to evaluate both QSAR and read-across generated data. Overall, decision trees, scoring schemes, and grouping or clustering of chemicals or endpoints are examples of potential strategies to identify NERCs. In Figure 1, a decision tree is shown to demonstrate how new signals can be analyzed to categorize chemicals of potential concern.

Various data integration approaches have been developed to identify NERCs or prioritize chemicals, one example being the EWS (NormaNEWS) by the NORMAN network using, among others, NTS data. In this system, semi-quantitative data on environmental occurrence are obtained for suspected compounds by searching in digitally archived high-resolution mass spectrometry data (dsfp.norman-data.eu). A pilot of an EWS by the Swedish Chemicals Agency applied cut-off values for several anticipated hazard attributes, including considering the applicability domain [47]. The Swedish Chemicals Agency has also developed a strategy that combines patent information with effect predictions for different endocrine receptors to predict chemicals of potential human concern [76]. Another attempt to find NERCs is the annual screening by ECHA of registration dossiers covering both hazard profiles and exposure estimates (echa.europa.eu). The Danish EPA uses combinations of QSAR models for both self- and hazard-classification of chemicals (Danish EPA). Models of various kinds have also been used to identify chemicals as persistent, bioaccumulative, mobile, and toxic [77,78,79]. These approaches are, for example, used to derive lists of potentially hazardous emerging chemicals for suspect screening activities [80,81].

**Figure 2:**
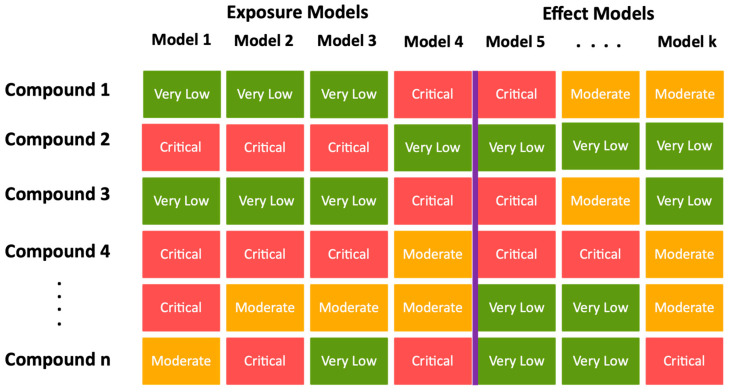
An example of a scoring matrix with a few predicted hazard properties and proposed model platforms used to detect NERCs within the EWS, with red suggesting an alarming property, orange suggesting a hazardous property is likely, and green indicating “safe”. The purple line separates exposure and effect models.

Another example of using and integrating data from multiple models is the scoring system developed by Hartmann et al., reaching a final score from 0 to 1 by assigning varying weights to various endpoints and structural alerts [82]. An alternative scoring system, open for use, is JANUS, which provides both single hazard property (e.g., persistence) scores and combined scores (e.g., substances of very high concern) (vegahub.eu). Applying heat mapping is a simple way to aggregate and score data from various sources (Figure 2). This method makes data ranking and visualization simple. It does, however, require defined parameter thresholds. To support decision-making, multiple criteria decision analysis (MCDA) encompasses a range of approaches that can handle multiple types of data at once, including quantitative, semi-quantitative, and qualitative data. Zheng et al. employed multiple hazard estimates to compare alternative brominated flame retardants [58]. Subsequently, their transformation products were also compared using MCDA. Like heat mapping, thresholds must still be set, but with MCDA, multiple data types are evaluated at once.

By grouping or clustering chemicals based on EWS data and chemical descriptors reflecting their structural and chemical characteristics, potentially hazardous chemicals with patterns resembling those of known pollutants can be identified using read-across approaches [83]. This can be facilitated using unsupervised machine learning techniques. Certain chemicals have a wealth of data and well-documented risks, so they could serve as positive controls or references when identifying NERCs with comparable descriptor patterns. Principal component analysis, hierarchical clustering, and k-nearest neighbor are examples of useful methods for this purpose [22].

## 6. Summary and Future Perspectives

**Figure 3:**
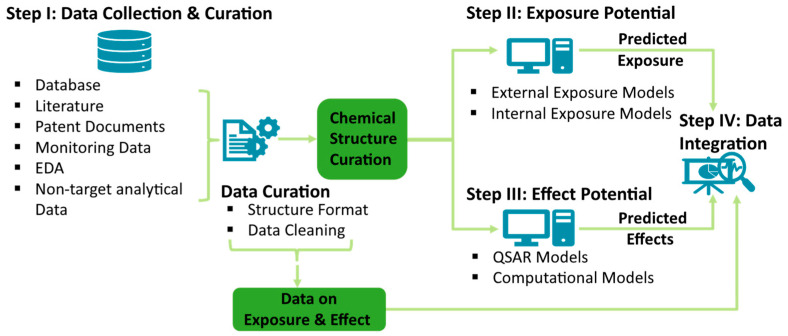
Proposed EWS workflow from (I) data collection and curation, (II) external and internal exposure modeling, and (III) effect modeling, to (IV) integration of signals for identification of potential NERCs.

With the recent developments in analytical chemistry, omics, and data science, signals of hazardous chemicals can be detected early. New screening techniques and tools can detect an unprecedented number of chemicals and their effects. Models can predict exposure and effects, while AI, NLP, and bioinformatic tools can handle massive amounts of diverse data. In light of the above-presented tools, the suggested EWS workflow can be used as a screening tool, particularly when there is a lack of data and even before chemicals are being commercialized. In Figure 3, a computational EWS is shown with data collection and signal curation to scoring exposure and effect potential, signal integration, and potential NERC notification. The EWS commences with the reporting of findings resulting from omics, non-target screening, or similar, or from a broad scope horizon scanning that is conducted regularly. Such scanning activities should cover grey literature, patent documents, environmental and human samples, and products and materials (Part I). Using NLP techniques and data curation methods will be essential to yield chemical structure information for use in the subsequent steps. NLP can also assist in automatic data collection monitoring, surveillance of global databases, and web scraping, and thus potentially detect, e.g., anomalies in real-time data. The methodologies have been used for the development of AOPs [84] and QSAR models [85], and to encode chemical structures and similarities [86].

The entry step of the EWS might go straight to data integration and scoring if enough experimental data for exposure and hazard scoring were identified. Alternatively, a curated chemical structure is the primary result from the data collection and curation phase (Step I), as an entry to external and internal exposure modeling (Step II) and effect modeling (Step III) (Figure 3). Exposure can be assessed in silico by predicting a compound’s potential fate, such as accumulation in biota or specific tissues, i.e., dose at target. Predicting the exposure potential will necessitate scenario settings for emissions, consumption and manufacturing information, and potential transformation reactions and their kinetics. Effect models should cover a wide range of effects, species, and biological complexity. Overall, several computational tools are available that can be integrated into an EWS to assess the exposure and effect potential. In addition, AI-based methodologies are being introduced in the field, providing an opening for better use of available data, and potentially improved predictive capacity for regulatory use [87]. Examples of applications include machine learning models developed for predicting toxicity of per- and polyfluoroalkyl substances [88], the use of transformers for structure decoding combined with deep learning to predict aquatic toxicity [89], and using neuronal networks to predict bioavailability [90]. Signals from the experimental data and computer models can be combined to form a matrix of exposure and effect indicators. Each compound can be scored based on its potential hazard properties, reliability, or other criteria (Step IV). This last step includes the integration of data that may lead to signals indicating a potential NERC that should be communicated to stakeholders where decisions are taken on next steps.

Despite these promising developments in data and model generation, combining them into an efficient EWS for identifying emerging issues is still a challenge for scientists and stakeholders. For example, many computational tools rely on experimental data and their applicability domains may not accommodate certain types of NERCs. It is also critical to develop experimental and computational models for less-studied health impacts including effects on the immune system, early neurodevelopment, and the metabolic system. In addition, transformation products and mixtures are frequently overlooked, as are certain types of compounds, such as polymers. Furthermore, the actual integration of EWS results, such as the development of a scoring system, presents a significant challenge in determining critical hazard levels. Finally, an EWS should ideally be built on a computational platform that is both maintainable and implementable while remaining user-friendly and in compliance with the FAIR principles. Furthermore, it should allow for automatization to alert responsible stakeholders as signals are identified. That would require seamless communication between different existing models and platforms. In conclusion, developing an EWS with a strong computational component would be a significant step toward the early detection and thus better assessment and mitigation of chemical risks.

## Data Availability

Not data is available for this publication to be shared.
